# Low education is associated with poor periodontal status in patients with type 2 diabetes mellitus: A cross‐sectional study

**DOI:** 10.1002/cre2.363

**Published:** 2020-11-30

**Authors:** Tatsuo Yamamoto, Michio Tanaka, Nobuichi Kuribayashi, Fuminobu Okuguchi, Haruhiko Isotani, Masahiro Iwamoto, Hidekatsu Sugimoto, Osamu Nakagawa, Masato Minabe, Shinya Fuchida, Yuki Mochida, Hiroki Yokoyama

**Affiliations:** ^1^ Department of Disaster Medicine and Dental Sociology, Graduate School of Dentistry Kanagawa Dental University Yokosuka Japan; ^2^ Misaki Naika Clinic Funabashi Japan; ^3^ Okuguchi Clinic of Internal Medicine Sendai Japan; ^4^ Isotani Diabetes Clinic Hirakata Japan; ^5^ Iwamoto Internal Medicine Clinic Zentsuji Japan; ^6^ Sugimoto Clinic Kitakyushu Japan; ^7^ Nakagawa Internal Medicine Clinic Sanjo Japan; ^8^ Department of Oral Interdisciplinary Medicine, Graduate School of Dentistry Kanagawa Dental University Yokosuka Japan; ^9^ Internal Medicine, Jiyugaoka Medical Clinic Obihiro Japan

**Keywords:** education, income, periodontal disease, type 2 diabetes mellitus

## Abstract

**Objectives:**

Cardiovascular disease remains the most common cause of death in patients with type 2 diabetes mellitus. Because periodontitis is a risk factor of cardiovascular disease, identification of risk factors of periodontitis is valuable to control periodontitis effectively. The purpose of this study was to examine the association of education and household income with periodontal status in patients with type 2 diabetes mellitus.

**Methods:**

Participants were 2,436 patients (59.8% male, aged 29–93 years) with type 2 diabetes mellitus from 27 medical clinics. Participants' medical records and information about education, household income, general health status, and health behaviors were collected. Periodontal status was assessed in a nearby dental office. Multiple linear regression analyses and ordered logistic regression analyses were conducted to examine the association of periodontal parameters with education and household income after adjusting for age, sex, general health status, and health behaviors.

**Results:**

Multiple linear regression analysis showed that mean probing pocket depth was not significantly associated with education and household income. Ordered logistic regression analyses showed statistically significant odds ratios (ORs) of junior high school (reference: university) for the tertiles of the percentage of sites with bleeding on probing (OR: 1.42; 95% confidence interval [CI]: 1.11–1.81), percentage of mobile teeth (OR: 1.58; 95% CI: 1.24–2.03), and number of teeth present (OR: 0.51; 95% CI: 0.39–0.65), and statistically significant odds ratios of high school (reference: university) for the tertiles of the percentage of mobile teeth (OR: 1.27; 95% CI: 1.06–1.51) and number of teeth present (OR: 0.74; 95% CI: 0.62–0.88), but not household income.

**Conclusions:**

These results suggest that low education is one of the important predictors of poor periodontal status in patients with type 2 diabetes mellitus. It is important to provide targeted interventions including periodontal education in junior high school.

## 1 INTRODUCTION

Cardiovascular disease (CVD) is the most prevalent cause of mortality and morbidity in patients with type 2 diabetes mellitus (T2DM) (Matheus et al., 2013). Subjects with T2DM exhibit a two to three‐fold higher risk of CVD than individuals without T2DM (Emerging Risk Factors Collaboration, 2010). Periodontitis has been epidemiologically indicated to play a causal role in the onset of CVD in the general population (Blaizot et al., 2009) and in Pima Indians with T2DM (Saremi et al., 2005).

Periodontitis is more prevalent in individuals with T2DM than in nondiabetic individuals (Mohamed et al., 2013; Shlossman et al., 1990) and is recognized as the sixth most common complication of T2DM (Loe, 1993). Therefore, maintaining periodontal health is important to reduce risk for cardiovascular disease in patients with T2DM.

To maintain periodontal health in T2DM patients, it is valuable to identify the factors associated with periodontitis in the population. Our previous pilot study of 503 subjects with T2DM from one medical clinic showed that education is strongly associated with periodontal parameters (Yokoyama et al., 2018).

Socioeconomic status (SES) is an important determinant of periodontal disease (Clarke & Hirsch, 1995). Cross‐sectional studies using general populations showed that low income and low education were associated with periodontitis in the United States (Borrell et al., 2006) and South Korea (Kim et al., 2014). A systematic review including eight longitudinal studies from countries other than Japan using general populations showed that relatively low SES earlier in life was associated with poorer periodontal health in adulthood (Schuch et al., 2017).

However, few studies have examined the association between SES and periodontal disease in Japan. Japan is known to have a less marked socioeconomic differential compared with other developed countries, and has had a universal health insurance system, including dentistry, since 1961. Only two studies using Japanese workers have shown association between occupational status and periodontal disease (Irie et al., 2017; Morita et al., 2007).

Few studies have examined the association between SES, especially education and income, and periodontal disease in T2DM patients in Japan. Information on the association between SES and periodontal status in T2DM patients is useful to target populations to improve periodontal health in such patients because of the bidirectional association (Taylor et al., 2013) and to improve collaboration between medical and dental healthcare providers to treat both T2DM and periodontal disease effectively (Sanz et al., 2018). Because subjects with T2DM have relatively lower SES than those without T2DM (Agardh et al., 2011), there might be no association between SES and periodontal status. Recently, both the prevalence of T2DM and socioeconomic inequality has increased in Japan (Chan et al., 2009; Kondo, 2012).

Therefore, the purpose of this study was to identify the association between SES and periodontal status among T2DM patients extending our previous pilot study (Yokoyama et al., 2018) using an increased number of patients from 27 medical clinics in Japan.

## 2 SUBJECTS AND METHODS

### 2.1 Study population

Participants were 2,436 patients (1,457 males and 979 females) aged 29–93 years (mean ± standard deviation: 66.9 ± 10.1 years) with T2DM who visited 27 Japanese medical clinics between December 2014 and March 2018. Healthcare providers at all 27 medical clinics belong to the Japan Clinicians Diabetes Association, and clinics were located in different areas throughout Japan. Patients visiting the medical clinics had already completed initial treatment for T2DM with an average duration of diabetes of 9.7 years (standard deviation: 6.6 years). T2DM was diagnosed according to the Japan Diabetes Society criteria: fasting blood glucose of ≥7.0 mmol/L, casual blood glucose of ≥11.1 mmol/L, or glycosylated hemoglobin A1c (HbA1c) of ≥6.5%. Diabetic subjects were treated with the aim of achieving the targets recommended by the Japan Diabetes Society (Haneda et al., 2018): HbA1c of <7.0%, blood pressure of <130/80 mmHg, and serum concentrations of low‐density lipoprotein cholesterol of <3.1 mmol/L, high‐density lipoprotein (HDL) cholesterol of ≥1.0 mmol/L, and non‐HDL cholesterol of <3.8 mmol/L.

Patients with no teeth; those who experienced acute myocardial infarction or cerebrovascular disease within the past 6 months; those with unstable angina pectoris, severe heart disease such as heart failure, cardiomyopathy, and valvular disease, severe liver diseases, cancer, or dementia; or those scheduled to undergo surgery were excluded. Patients with missing data for any of the parameters evaluated in the present study were also excluded.

The study protocol was approved by the ethics committees of Jiyugaoka Medical Clinic (October 27, 2014, No. 250716) and Kanagawa Dental University (July 5, 2016, No. 387; September 12, 2017, No. 446; December 20, 2017, No. 476). All participants provided written informed consent, and the study was carried out in accordance with the revised Declaration of Helsinki.

### 2.2 Periodontal examination

Subjects were asked to visit a nearby dental office with a uniform dental chart to receive a periodontal examination, irrespective of whether they were attending the dental office regularly (Tanaka et al., 2019; Yokoyama et al., 2018). Subjects were informed that the periodontal examination was conducted under healthcare services covered by their health insurance. In addition, subjects were asked to hand a letter to the dentists. The letter provided an explanation of the present study including the background, purpose, and methods and requested that the dentists send the patient's dental chart to the medical clinics by mail.

The dental chart included probing pocket depth (PPD), bleeding on probing (BOP) (Lang et al., 1990), tooth mobility (Miller, 1950), and number of teeth present. PPD was defined as the distance from the gingival margin to the base of the clinical periodontal pocket and was measured at six sites per tooth (mesio‐buccal, mid‐buccal, disto‐buccal, mesio‐lingual/palatal, mid‐lingual/palatal, and disto‐lingual/palatal). BOP for each tooth was recorded as present if it occurred in at least one of the six sites of each tooth within 30 seconds of probing. Manual examination of tooth mobility was assessed as yes/no.

### 2.3 General health status

HbA1c was measured by high‐performance liquid chromatography, which has been certified by the American National Glycohemoglobin Standardization Program. Blood pressure was measured by an appropriately sized cuff using an automated blood pressure device. The height and weight of the participants were measured, and body mass index (BMI) was calculated as the weight divided by the square of the height (kilograms per square meter). Use of calcium antagonists, treatment of diabetes and duration of diabetes were obtained from patients' records. Regarding treatment of diabetes, subjects were divided into groups by treatment with diet alone, hypoglycemic tablets, or insulin.

### 2.4 Socioeconomic status and health behaviors

Information about education, household income, smoking, and toothbrushing frequency was obtained using a questionnaire (Tanaka et al., 2019; Yokoyama et al., 2018). Education was categorized as junior high school, high school, and university. Junior college was included in the category of university. Household income was categorized as <3.00 million, 3.00–4.99 million, and ≥5.00 million Japanese yen (100 Japanese yen = 1 US dollar, in 2018) per year. Smoking was categorized as never, ex, and current. Toothbrushing frequency was categorized as once, twice, and ≥3 times per day.

### 2.5 Statistical analysis

Mean PPD, percentage of BOP‐positive teeth, and percentage of mobile teeth were calculated for each subject. Because distributions of the percentage of BOP‐positive teeth, percentage of mobile teeth, and number of teeth present were skewed, we divided the data into tertiles considering the parallel regression assumption in the ordered logistic regression (Murayama et al., 2020).

Continuous variables such as age, HbA1c, systolic blood pressure, BMI, duration of diabetes and mean PPD were compared among three education groups or three household income groups using one‐way analysis of variance. Categorical variables such as sex, smoking, use of calcium antagonists, toothbrushing frequency, and three categories of the percentage of BOP, percentage of mobile teeth, and number of teeth present were compared among three education groups or three household income groups using the chi‐squared test. Association between education and household income was also examined using the chi‐squared test.

We fit multiple linear regression models for mean PPD and ordered logistic regression models for three categories of the percentage of BOP, percentage of mobile teeth, and number of teeth present to examine the association between socioeconomic status (education and household income) and periodontal parameters. In Model 1, dummy variables of junior high school, high school, household income of <3.00 million Japanese yen, and household income of 3.00–4.99 million Japanese yen, sex, and age were added. The dummy variable is a variable that takes on the value 0 or 1. In Model 2, systemic condition such as HbA1c, systolic blood pressure, BMI, use of calcium antagonists, diabetes therapy (dummy variables of diet alone and hypoglycemic tablets) and duration of diabetes were added to Model 1. In Model 3, health behaviors such as smoking (dummy variables of never and current) and toothbrushing frequency (dummy variables of once per day and twice per day) were added to Model 2. Dummy variables were selected considering multicollinearity using variance inflation factors. We confirmed the parallel regression assumption in the ordered logistic regression models. The estimation for ordered logistic regression models was presented as odds ratios (ORs) with 95% confidence intervals (CIs). All statistical analyses were performed using IBM SPSS Statistics (version 24.0; IBM Co., New York, NY).

## 3 RESULTS

The characteristics of participants according to education are shown in Table 1. Subjects with lower education tended to be older, female, have lower BMI, were never smokers, used calcium antagonists, had longer duration of diabetes, had lower household income, had a greater number of mobile teeth, and had fewer number of teeth present compared to those with higher education.

**TABLE 1:** Characteristics of participants according to education

Variables	Education			
	Junior high school	High school	University	*p*
	*n* = 401	*n* = 1,256	*n* = 779	
Age, years (mean and SD)	72.7 (8.5)	66.9 (9.8)	64.0 (10.1)	<.001^a^
Male sex, %	46.4	55.7	73.3	<.001^b^
HbA1c, % (mean and SD)	7.0 (0.8)	7.0 (0.8)	7.0 (0.8)	.469^a^
Systolic blood pressure, mmHg (mean and SD)	127 (13)	126 (14)	126 (13)	.186^a^
Body mass index, kg/m^2^ (mean and SD)	24.9 (4.0)	24.7 (4.0)	25.2 (3.9)	.023^a^
Smoking, %				
Never	62.3	53.5	49.4	<.001^b^
Past	29.2	31.5	35.3	
Current	8.5	15.0	15.3	
Use of calcium antagonists, %	37.9	31.9	27.3	.001^b^
Therapy, %				
Diet	9.5	9.1	10.0	.809^b^
Tablet	71.6	73.4	73.6	
Insulin	19.0	17.5	16.4	
Duration of diabetes	11.4 (7.5)	9.8 (6.6)	8.7 (5.9)	<.001^a^
Toothbrushing frequency, per day, %				
Once	22.9	19.5	20.9	.208^b^
Twice	47.4	54.2	52.4	
Three times or more	29.7	26.3	26.7	
Household income, million JPY, %				
<3.00	71.6	47.7	27.2	<.001^b^
3.00–4.99	19.5	34.0	32.2	
≥5.00	9.0	18.3	40.6	
Mean PPD, mm (mean and SD)	2.8 (0.8)	2.8 (0.8)	2.8 (0.7)	.323^a^
Percentage of sites with BOP				
<19.3 (*n* = 819)	31.7	33.2	35.3	.201^b^
12.3–53.6 (*n* = 810)	30.4	34.3	33.0	
≥53.7 (*n* = 807)	37.9	32.5	31.7	
Percentage of mobile teeth				
0 (*n* = 1,014)	31.2	40.9	48.1	<.001^b^
0.01–17.24 (*n* = 610)	24.9	24.1	26.6	
≥17.25 (*n* = 812)	43.9	35.0	25.3	
Number of teeth present				
<22 (*n* = 863)	54.1	36.3	24.4	<.001^b^
22–26 (*n* = 790)	27.4	33.4	33.5	
≥ 27 (*n* = 783)	18.5	30.3	42.1	

Abbreviations: BOP, bleeding on probing; HbA1c, glycated hemoglobin A1c; PPD, probing pocket depth, SD, standard deviation.

^a^ One‐way analysis of variance.

^b^ Chi‐squared test.

The characteristics of participants according to household income are shown in Table 2. Subjects with lower household income tended to be older, female, had lower BMI, were never smokers, had longer duration of diabetes, had lower frequency of toothbrushing, had a greater number of mobile teeth, and had fewer number of teeth present compared to those with higher household income.

**TABLE 2:** Characteristics of participants according to household income

Variables	Household income (million JPY)			
	Less than 3.00	3.00–4.99	5.00 or more	*p*
	*n* = 1,098	*n* = 756	*n* = 582	
Age, years (mean and SD)	70.2 (8.9)	66.5 (9.9)	61.4 (9.9)	<.001^a^
Male sex, %	52.4	61.8	71.3	<.001^b^
HbA1c, % (mean and SD)	7.0 (0.8)	7.0 (0.8)	7.0 (0.9)	.484^a^
Systolic blood pressure, mmHg (mean and SD)	126 (14)	127 (13)	126 (13)	.580^a^
Body mass index, kg/m^2^ (mean and SD)	24.7 (3.9)	24.7 (3.9)	25.7 (3.9)	<.001^a^
Smoking, %				
Never	59.7	52.5	43.6	<.001^b^
Past	28.4	35.8	35.2	
Current	11.8	11.6	21.1	
Use of calcium antagonists, %	33.9	30.2	28.5	.052^b^
Therapy, %				
Diet	10.3	9.3	8.1	.094^b^
Tablet	71.5	72.2	77.5	
Insulin	18.2	18.5	14.4	
Duration of diabetes	10.6 (6.8)	9.7 (6.6)	8.2 (5.9)	<.001^a^
Toothbrushing frequency, per day, %				
Once	23.2	17.7	19.1	.012^b^
Twice	49.1	56.5	53.8	
Three times or more	27.7	25.8	27.1	
Mean PPD, mm (mean and SD)	2.8 (0.7)	2.8 (0.8)	2.8 (0.8)	.503^a^
Percentage of sites with BOP				
<19.3 (*n* = 819)	33.5	35.7	31.1	.426^b^
12.3–53.6 (*n* = 810)	32.7	33.2	34.4	
≥53.7 (*n* = 807)	33.8	31.1	34.5	
Percentage of mobile teeth				
0 (*n* = 1,014)	37.4	43.5	47.1	.001^b^
0.01–17.24 (*n* = 610)	25.6	24.7	24.4	
≥17.25 (*n* = 812)	37.0	31.7	28.5	
Number of teeth present				
<22 (*n* = 863)	43.3	32.8	24.1	<.001^b^
22–26 (*n* = 790)	30.8	34.0	33.5	
≥27 (*n* = 783)	26.0	33.2	42.4	

Abbreviations: BOP, bleeding on probing; HbA1c, glycated hemoglobin A1c; PPD, probing pocket depth, SD, standard deviation.

^a^ One‐way analysis of variance.

^b^ Chi‐squared test.

Mean PPD values were not significantly different by groups of education and household incomes in Tables 1 and 2. However, age and sex differences were prominent among groups of education and household incomes. Therefore, the associations of PPD values with education and household incomes were analyzed after adjusting for age and sex.

Results of multiple linear regression analyses for mean PPD are shown in Table 3. Mean PPD was significantly and positively correlated with junior high school (*β*: .107) and high school (*β*: .074) in Model 1 (*p* < .05) and positively correlated with high school (*β*: .070) in Model 2 (*p* < .05); however, the association was no longer significant in Model 3. Additionally, mean PPD was not significantly associated with household income.

**TABLE 3:** Multiple linear regression models for mean probing pocket depth

Variables	Model 1				Model 2				Model 3			
	Non‐standardized		Standardized *β*	*p*	Non‐standardized		Standardized *β*	*p*	Non‐standardized		Standardized *β*	*p*
	B	SE			B	SE			B	SE		
Education (reference: University)												
Junior high school	0.107	0.050	.053	.032	0.089	0.050	.044	.074	0.082	0.050	.041	.098
High school	0.074	0.036	.049	.038	0.070	0.035	.047	.047	0.059	0.035	.039	.098
Household income, Million JPY (reference: ≥ 5.00)												
<3.00	0.004	0.042	.003	.929	0.015	0.042	.010	.732	0.021	0.042	.014	.623
3.00–4.99	−0.036	0.043	−.022	.392	−0.027	0.042	−.016	.530	−0.018	0.042	−.011	.664

*Note*: Model 1: adjusted for age and sex. Model 2: Model 1 + HbA1c, systolic blood pressure, body mass index, use of calcium antagonists, diabetes therapy and duration of diabetes. Model 3: Model 2 + smoking and toothbrushing frequency.

Abbreviation: SE, standard error.

The associations of education and household incomes with BOP, tooth mobility, and tooth number were analyzed by ordered logistic regression because these variables had skewed distribution. Results of ordered regression analyses for tertiles of the percentage of sites with BOP, percentage of mobile teeth, and number of teeth present are shown in Table 4. All three parameters were significantly associated with junior high school in Models 1–3 (*p* < .01). ORs (95% CIs) of junior high school (reference: university) in Model 3 for the percentage of BOP, percentage of mobile teeth, and number of teeth present were 1.42 (1.11–1.81), 1.58 (1.24–2.03), and 0.51 (0.39–0.65), respectively, with statistical significance (*p* < .01). ORs (95% CIs) of high school (reference: university) in Model 3 for the percentage of mobile teeth and number of teeth present were 1.27 (1.06–1.51) and 0.74 (0.62–0.88), respectively, with statistical significance (*p* < .05).

**TABLE 4:** Ordered logistic regression for tertiles of the percentage of bleeding on probing, percentage of mobile teeth, and number of teeth present

Dependent variables	Independent variables	Model 1				Model 2				Model 3			
		OR	95% CI		*p*	OR	95% CI		*p*	OR	95% CI		*p*
Percentage Of BOP	Education (reference: University)												
	Junior high school	1.41	1.11	1.80	.005	1.42	1.11	1.81	.005	1.42	1.11	1.81	.005
	High school	1.13	0.95	1.34	.176	1.13	0.95	1.35	.161	1.13	0.95	1.34	.172
	Household income, million JPY (reference: ≥ 5.00)												
	<3.00	0.96	0.79	1.18	.723	1.01	0.82	1.24	.942	0.99	0.80	1.22	.908
	3.00–4.99	0.86	0.70	1.06	.164	0.89	0.72	1.10	.277	0.89	0.72	1.09	.262
Percentage of mobile teeth	Education (reference: University)												
	Junior high school	1.64	1.29	2.09	<.001	1.62	1.27	2.07	<.001	1.58	1.24	2.03	<.001
	High school	1.30	1.09	1.55	.003	1.31	1.10	1.56	.003	1.27	1.06	1.51	.009
	Household income, million JPY (reference: ≥ 5.00)												
	<3.00	0.99	0.81	1.22	.942	0.99	0.81	1.23	.959	1.02	0.83	1.26	.855
	3.00–4.99	0.93	0.75	1.15	.498	0.94	0.76	1.17	.590	0.97	0.78	1.19	.756
Number of teeth present	Education (reference: University)												
	Junior high school	0.47	0.36	0.60	<.001	0.48	0.38	0.62	<.001	0.51	0.39	0.65	<.001
	High school	0.70	0.59	0.84	<.001	0.70	0.59	0.84	<.001	0.74	0.62	0.88	.001
	Household income, million JPY (reference: ≥ 5.00)												
	<3.00	0.85	0.69	1.05	.129	0.85	0.69	1.05	.140	0.81	0.66	1.00	.053
	3.00–4.99	0.96	0.78	1.19	.741	0.96	0.78	1.19	.709	0.91	0.73	1.13	.382

*Note*: Model 1: adjusted for age and sex. Model 2: Model 1 + HbA1c, systolic blood pressure, body mass index, use of calcium antagonists, diabetes therapy and duration of diabetes. Model 3: Model 2 + smoking and toothbrushing frequency.

BOP: bleeding on probing, CI: confidence interval, OR: odds ratio.

## 4 DISCUSSION

The results of the present study showed that T2DM patients with lower education have poorer periodontal health and lower number of teeth present than those with higher education after adjusting for possible confounders. These results extended the results of our previous pilot study (Yokoyama et al., 2018) and further found that, in a large number of patients with T2DM seen in 27 outpatient clinics throughout Japan, junior high school education was associated with poorer periodontal health including higher percentage of sites with BOP, higher number of mobile teeth, and lower number of teeth present than those with university education after adjusting for possible confounders. These results underline the importance of periodontal education in junior high school.

These results of the present study agree with the following previous studies using general populations. An association between low education and periodontitis has been reported using pooled data of general populations of the United States, Australia, Iran, Denmark, Taiwan, Canada, Sweden, Brazil, and Thailand (Boillot et al., 2011). Moreover, an association between low education and lower number of teeth present has been reported in the Japanese general population (Ueno et al., 2012).

Statues of diabetes mellitus of the patients were well controlled because mean (standard deviation) of HbA1c level was 7.0 (0.8). This means that the effects of diabetes mellitus on periodontal tissue were weak. In fact, HbA1c level was significantly associated with mean PPD and percentage of BOP but not percentage of mobile teeth and number of teeth present in the fully adjusted Model 3 (data not shown). These results suggest that education is a risk factors of periodontitis in Japanese T2DM patients.

However, lack of an association of household income with periodontal status and number of teeth present in the present study disagreed with the results from previous studies using general populations of United Status (Borrell et al., 2006) and South Korea (Kim et al., 2014). One potential reason for this inconsistency may be that we enrolled only T2DM patients and most subjects were older, retired, and pensioners. In addition, all subjects in the present study visited dentists to receive oral examination, which may have resulted in the unintended exclusion of subjects with lower economic status because people with low economic status generally tend to not visit dentists (Yamamoto, Kondo, Aida, Suzuki, et al., 2014).

Periodontal disease may develop in a way that deepening periodontal pocket progresses to tooth mobility, finally leading to tooth loss. PPD was deeper in patients with low education after adjusting for age and sex in our study. In the fully adjusted Model 3, percentage of mobile teeth, but not mean PPD, was associated with education. The results suggest that severe periodontitis is especially associated with education in this study population because tooth mobility reflects severe periodontitis in general (Miller, 1950). The explanation agrees with the finding that the prevalence of periodontitis in our study cohort was higher than that in the general population in Japan (Ministry of Health, Labour and Welrare, 2016). Percentages of subjects in the present study with PPD of 4 mm or deeper aged in their 30s, 40s, 50s, 60s, 70s, and 80s or older in the present study were 88%, 86%, 89%, 90%, 90%, and 90%, respectively, which were higher than those in the general population in Japan excluding edentulous subjects (37%, 45%, 52%, 64%, 66%, and 74%, respectively). Moreover, percentages of subjects in the present study with PPD of 6 mm or deeper aged in their 30s, 40s, 50s, 60s, 70s, and 80s or older in the present study were 42%, 41%, 49%, 54%, 49%, and 55%, respectively, which were higher than those in the general population in Japan excluding edentulous subjects (5%, 5%, 10%, 17%, 14%, and 12%, respectively).

The possible pathways underlying superior periodontal health observed in university graduates among T2DM patients may be related to many factors including general health status and health behaviors. The ORs of junior high school for percentage of mobile teeth and the number of teeth present were slightly attenuated by adding general health status and health behaviors from Models 1, 2, and 3. Because obesity (Wilkins et al., 2017), smoking (Zini et al., 2011), and toothbrushing (Clarke & Hirsch, 1995) are well‐known risk factors for periodontal disease, these factors might mediate the association. However, after adjusting for general health status and health behaviors, significant associations between education and periodontal health and number of teeth present were observed in Model 3. This indicates the presence of other possible mediating factors, which may include job and dental attendance (Holde et al., 2018; Irie et al., 2017; Morita et al., 2007; Yamamoto, Kondo, Aida, Fuchida, et al., 2014). Further studies are required to confirm the possible pathway including longitudinal design from a life‐course perspective (Schuch et al., 2015).

Our study has some strengths and limitations. First, the sample size of the present study is relatively large and data were obtained from 27 medical clinics. Therefore, the results from the present study could be, in part, generalized to T2DM patients in Japan. Second, six sites of all teeth were examined when periodontal pocket probing was performed. However, periodontal probing was not calibrated and dentist‐to‐dentist variations were not evaluated because the study was performed in a community‐based primary care setting. Third, the inspection equipment and measurement environment were not necessarily unified among medical and dental clinics. Forth, the selection bias for subjects could be arisen in the method because attending the dental offices were required for the subjects in the present study. Fifth, information on use of interdental brush and regular dental visit, which might be useful to explain the association between SES and periodontal parameters, were not available.

Sixth, the age range of the participants was wide, ranging from 29 to 92 years old. Because education has undergone the changes such as a rise in the entrance ratio to high school and university in Japan, older people tend to be less educated and to have more periodontal disease than younger people. Stratified analyses by age categories (<65 year‐old and ≥65) were conducted and revealed that junior high school education was associated with poorer periodontal health including higher percentage of sites with BOP, higher number of mobile teeth, and lower number of teeth present than those with university education after adjusting for possible confounders (data not shown).

## 5 CONCLUSIONS

This cross‐sectional study with a large number of T2DM patients seen in medical clinics throughout Japan showed that lower education is associated with poorer periodontal status and lower number of teeth present after adjusting for possible confounders. There was no significant association between household income and periodontal status and number of teeth present. These results suggest the importance of periodontal education in junior high school.

## CONFLICT OF INTEREST

The authors declare that they have no conflict of interest.

## Data Availability

The data that support the findings of this study are available on request from the corresponding author. The data are not publicly available due to privacy or ethical restrictions.
